# Exogenous protease supplementation in high- and low-fishmeal diets for Pacific white shrimp (*Penaeus vannamei*): Comparative effect on growth, immunity, nutrient digestibility and gut health

**DOI:** 10.1016/j.aninu.2025.04.002

**Published:** 2025-05-09

**Authors:** Mirasha Hasanthi, Rutchanee Chotikachinda, Nalin Medagoda, Kyeong-Jun Lee

**Affiliations:** a Department of Marine Life Science, Jeju National University, Jeju 63243, South Korea; b DSM Nutritional Products Ltd., Bangkok 10310, Thailand; c Marine Life Research Institute, Jeju National University, Jeju 63333, South Korea

**Keywords:** Protease, Enzyme, Pacific white shrimp, Aquafeed, Protein, Digestibility

## Abstract

The present study evaluated the effects of exogenous protease supplementation in low- and high-fishmeal (FM) diets on growth performance, feed utilization, innate immunity, digestive enzyme activity, nutrient digestibility, gut microbiota and intestinal morphology of Pacific white shrimp (*Penaeus vannamei*). A 2 × 3 factorial experiment was designed with two dietary FM levels (200 g/kg, positive control [PC]; and 100 g/kg, negative control [NC]) and three protease levels (0, 400 and 800 mg/kg) to obtain six experimental diets designated as PC, PC400, PC800, NC, NC400, and NC800. Six replicate groups of 30 shrimp (initial weight 0.30 ± 0.01 g) were fed the diets for 8 wk. Protease supplementation significantly improved (*P* < 0.001) growth performance and feed utilization efficiency in both high- and low-FM diets. Innate immunity and antioxidant enzyme activities were significantly enhanced (*P* < 0.001) with increasing FM and protease levels. Furthermore, the inclusion of protease in low-FM diets significantly increased (*P* < 0.001) total hemocyte count and phagocytic, phenoloxidase, lysozyme and superoxide dismutase activities, reaching levels comparable to the PC group. Increasing FM and protease levels significantly upregulated (*P* < 0.001) the expression of *proPO*, crustin, *TGF-β*, *LvIKK-β* and *TLR3* genes, while downregulating *TNF-α*. The inclusion of protease in the low FM diet significantly increased (*P* < 0.05) digestive enzyme activities, intestinal villi length, whole-body amino acid composition and nutrient digestibility to the levels comparable to the PC group. The relative abundance of heterotrophic marine bacteria (*P* < 0.001), Gram-positive bacteria (*P* = 0.034) and *Lactobacilli* spp. (*P* < 0.001) in the gut significantly increased (*P* < 0.05) with increasing protease levels, while an inverse relationship was observed for *Vibrio* spp. (*P* < 0.001). These results demonstrated that protease supplementation in either the high- or low-FM diets could improve shrimp growth, feed utilization efficiency, immunity, nutrient digestibility, intestinal morphology and gut microbiome. Notably, supplementing the low-FM diet with 800 mg/kg protease improved shrimp performance, reaching levels comparable to those obtained with the PC diet.

## 1 Introduction

The rapid expansion of the global aquaculture industry has significantly increased the demand for fishmeal (FM) due to its longstanding use as the main dietary protein source in aquafeed (Tacon and Metian, 2008). However, the high price and scarcity of FM demonstrate the increasing difficulty to meet the growing demand (Luthada-Raswiswi et al., 2021). Continuous efforts have been made to minimize FM inclusion levels by exploring various alternative protein sources such as plant-based proteins, terrestrial animal and fishery by-products, insect proteins and single cell proteins derived from bacteria, algae and yeast (Gasco et al., 2018; Gatlin et al., 2007; Hardy, 2010; Villanueva-Gutiérrez et al., 2020). However, a high level of substitution of FM remains a challenge and is associated with poor performance, as these protein sources often contain imbalanced amino acid profiles and anti-nutritional factors (ANFs) that adversely affect gut integrity and immune function (Oliva-Teles et al., 2015; Thakur et al., 2019). For example, plant-derived protein sources contain various ANFs, including tannins, phytic acid, saponins, protease inhibitors, trypsin inhibitors, lectins, gossypol, and non-starch polysaccharides (Ali et al., 2022). These substances often combine with other nutrients, thereby reducing their digestibility and bioavailability. Cereals and legumes primarily contain trypsin inhibitors, protease inhibitors and phytates, which negatively affect protein digestibility and mineral (P, Ca, Fe, Zn, Cu and Mg) absorption (Raes et al., 2014). Therefore, various strategies such as microbial fermentation (Dawood and Koshio, 2020), heat treatment (Laguna et al., 2017), supplementation of essential amino acids (Guo et al., 2020) and exogenous acidifiers (Ng and Koh, 2017) have been employed to reduce or eliminate ANFs and enhance feed quality.

The supplementation of digestive enzymes (protease, lipase, amylase, cellulase and hemicellulase) in feeds has been reported to decrease the effects of ANFs, increase digestibility and enhance nutrient bioavailability (Hlophe-Ginindza et al., 2016; Liang et al., 2022; Monier, 2020). The primary purpose of enzyme application in feeds is to improve digestion efficiency (Zheng et al., 2020). Proteolytic enzymes, also known as proteases, are integral components of all living organisms including animals, plants, algae, bacteria and viruses. These enzymes catalyze the breakdown proteins into smaller polypeptides, dipeptides or eventually into single amino acids, thereby playing a crucial role in protein catabolism and digestion by hydrolyzing peptide bonds (López-Otín and Bond, 2008). Exopeptidases hydrolyze peptide bonds to detach terminal amino acids from the substrate, while endopeptidases cleave the internal peptide bonds within a protein (Fu et al., 2021).

Proteases play a crucial role in protein digestion by facilitating nutrient availability and absorption through their proteolytic activity while reducing organic waste generation (Liang et al., 2022). Their functions extend beyond digestion, playing key roles in multiple biological processes. For instance, proteases regulate specific proteolytic reactions that lead to the production of new bioactive molecules, cellular information processing, cell proliferation and differentiation, DNA replication and transcription, tissue morphogenesis, blood coagulation, wound repair, immunity, inflammation, programmed cell death, gastrointestinal health, prohormone maturation as well as cellular protein recycling (López-Otín and Bond, 2008; Mótyán et al., 2013). Recent findings indicated that the incorporation of exogenous proteases as feed additives significantly enhanced growth, metabolic activity, feed utilization efficiency, gastrointestinal health, digestive enzyme activities and nutrient digestibility and retention in farm animals and aquatic species (Hlophe-Ginindza et al., 2016; Monier, 2020; Zuo et al., 2015). However, their potential application in invertebrate nutrition, particularly in shrimp feed, has received little attention (Divakaran and Velasco, 1999; Song et al., 2017). To date, no study has comparatively evaluated the use of protease across different FM levels in the shrimp diet. This study is the first to assess the impact of exogenous protease in both high- and low-FM diets for Pacific white shrimp (*Penaeus vannamei*) using comprehensive parameters.

The Pacific white shrimp is the most widely cultured crustacean species, accounting for approximately 51.7% of crustacean production worldwide (FAO, 2022). In crustaceans, the production of digestive enzymes is influenced by various factors including age, life stage, molting stage, feed consumption, circadian cycle and culture conditions (Perera et al., 2008; Veróonica and Gimenez, 2013). Certain digestive enzymes are deficient in aquatic animals, particularly in the early developmental stages (Biesiot and Capuzzo, 1990). Therefore, this study sought to investigate the effects of exogenous protease supplementation in high- and low-FM diets for juvenile Pacific white shrimp to reduce the adverse effects of plant protein ingredients.

## 2 Materials and methods

### 2.1 Animal ethics statement

All the experimental procedures applied in this study were reviewed and approved by the Animal Care and Use Committee of Jeju National University (approval number 2022-0044).

### 2.2 Experimental diets

A high FM (HFM) diet (200 g FM/kg) and a low FM (LFM) diet (100 g FM/kg) were formulated as a positive control (PC) and a negative control (NC) diet, respectively. In the LFM diet, 100 g/kg of FM was iso-nitrogenously replaced with soybean meal. Exogenous protease was supplemented to PC and NC diets at 0, 400 and 800 mg/kg levels to obtain six experimental diets (PC, PC400, PC800, NC, NC400 and NC800). The protease (81,800 U/g; a serine protease produced by submerged fermentation of *Bacillus licheniformis*) was supplied by DSM Nutritional Products Ltd., Bangkok, Thailand. All the diets were formulated to be isonitrogenous (38% crude protein) and isolipidic (8% crude lipid). Dietary ingredients were finely pulverized and measured according to the diet formulation. The protease was in granular form (500 μm average particle size) and incorporated with the dry ingredients. After completely mixing dry components with fish oil and distilled water (15%), moist doughs were pelleted into approximately 2 mm diameter pellets. During the pelleting process, the temperature was kept below 28 °C. Diets were dried at 25 °C for 8 h with an electric drier to reduce the moisture level to less than 10% and stored in a freezer at −24 °C until daily utilization. Following the feed production and 8 wk of storage during the feeding trial, all feed samples were sent to DSM technical analytical service to analyze protease activity and confirm whether the enzyme remained active throughout the study. One protease unit (U) is the amount of enzyme that releases 1 μmol of p-nitroaniline from 1 mmol/L of N-succinyl-Ala-Ala-Pro-Phe-p-nitroanilide (SAPNA) per minute at pH 9.0 and temperature at 37 °C. Diet formulation, analyzed proximate composition and enzyme activity are provided in Table 1. The dietary amino acid composition is presented in Table 2.

**Table 1:** Formulation and proximate composition of the experimental diets for Pacific white shrimp (*Penaeus vannamei*) (g/kg diet, DM basis).

Item	Experimental diets^7^					
	PC	PC400	PC800	NC	NC400	NC800
**Ingredients**						
Tuna FM^1^	100.00	100.00	100.00	100.00	100.00	100.00
Sardine FM^2^	100.00	100.00	100.00	0.00	0.00	0.00
Soybean meal^3^	333.34	333.34	333.34	465.63	465.63	465.63
Squid liver powder	50.00	50.00	50.00	70.00	70.00	70.00
Fish protein concentrate	5.00	5.00	5.00	5.00	5.00	5.00
L-Lysine HCl	2.62	2.62	2.62	3.16	3.16	3.16
Methionine	2.20	2.20	2.20	2.93	2.93	2.93
Taurine	0.18	0.18	0.18	0.25	0.25	0.25
Fish oil	6.71	6.71	6.71	10.94	10.94	10.94
Soy lecithin	30.00	30.00	30.00	27.24	27.24	27.24
Cholesterol	0.56	0.56	0.56	0.70	0.70	0.70
Choline chloride	10.00	10.00	10.00	10.59	10.59	10.59
Wheat floor	306.58	306.18	305.78	244.57	244.17	243.77
Wheat gluten meal	20.00	20.00	20.00	20.00	20.00	20.00
Monocalcium phosphate	16.81	16.81	16.81	22.99	22.99	22.99
Vitamin premix^4^	3.00	3.00	3.00	3.00	3.00	3.00
Mineral premix^5^	3.00	3.00	3.00	3.00	3.00	3.00
Carboxymethyl cellulose	10.00	10.00	10.00	10.00	10.00	10.00
Protease^6^	0.00	0.40	0.80	0.00	0.40	0.80
Total	1000.00	1000.00	1000.00	1000.00	1000.00	1000.00
**Proximate composition**						
Dry matter, fed basis	920.72	920.44	921.58	920.80	922.12	921.61
Organic matter	838.43	839.21	839.42	840.40	840.55	840.55
Crude protein	382.91	384.27	382.31	379.71	381.08	380.51
Crude lipid	80.56	83.01	81.65	82.23	82.31	81.52
Ash	82.29	81.23	82.16	80.40	81.57	81.06
Phosphorus	21.12	21.02	21.68	22.66	23.05	21.78
Protease activity, U/kg	0.00	38,565	64,200	0.00	38,172	64,320

PC = positive control; NC = negative control; FM = fishmeal.

1 Wooginfeed Industry Co. Ltd., Incheon, South Korea.

2 Triple Nine Fish Protein Co. Ltd., Lota, Chile.

3 CJ Cheiljedang Corp., Seoul, South Korea.

4 Vitamin premix (g/kg): retinol, 1.65; ergocalciferol, 0.025; tocopherol, 20.0; menadione, 5.00; ascorbic acid, 20.0; thiamine, 2.00; riboflavin, 20.0; pyridoxine, 15.0; folic acid, 4.00; cobalamin, 0.01; inositol, 54.0; nicotinic acid, 40.0; calcium pantothenate, 30.0 and biotin, 0.30.

5 Mineral premix (g/kg): MgSO_4_·7H_2_O, 80.0; NaH_2_PO_4_·2H_2_O, 370.0; KCl, 130.0; Ferric citrate, 40.0; ZnSO_4_·7H_2_O, 20.0; Ca-lactate, 356.5; CuCl_2_, 0.2; AlCl_3_·6H_2_O, 0.15; Na_2_Se_2_O_3_, 0.01; MnSO_4_·H_2_O, 2.0; CoCl_2_·6H_2_O, 1.0.

6 DSM Nutritional Products, Bangkok, Thailand.

7 PC, high fishmeal diet (200 g FM/kg); PC400, PC, diet supplemented with protease at 400 mg/kg; PC800, PC, diet supplemented with protease at 800 mg/kg; NC, low fishmeal diet (100 g FM/kg); NC400, NC, diet supplemented with protease at 400 mg/kg; NC800, NC, diet supplemented with protease at 800 mg/kg.

**Table 2:** Amino acid composition (g/kg diet, DM basis) of the six experimental diets for Pacific white shrimp (*Penaeus vannamei*).

Item	Experimental diets^1^					
	PC	PC400	PC800	NC	NC400	NC800
**Essential amino acids**						
Arginine	23.00	22.90	22.70	23.70	22.70	23.90
Threonine	13.50	13.80	13.60	12.80	12.90	13.30
Valine	16.30	16.20	16.10	15.70	15.90	16.00
Phenylalanine	15.60	15.80	15.60	15.60	15.40	15.70
Isoleucine	14.50	14.50	14.40	14.40	14.80	14.90
Leucine	24.70	24.90	24.50	24.00	24.10	23.70
Histidine	8.60	8.70	8.30	7.80	8.60	8.20
Lysine	18.10	18.20	18.40	16.30	17.10	17.00
Methionine	7.40	7.30	7.50	6.80	7.10	7.00
Taurine	1.00	1.10	1.00	0.50	0.50	0.50
**Non-essential amino acids**						
Aspartic acid	32.20	32.50	31.30	32.40	33.10	33.50
Alanine	17.50	17.70	17.40	15.80	17.40	16.30
Serine	16.10	16.40	15.70	15.80	15.50	16.70
Glutamic acid	73.30	73.60	73.10	72.20	74.50	74.60
Proline	19.30	19.10	21.10	15.80	16.60	16.90
Glycine	19.40	20.10	19.50	17.20	19.20	18.20
Tyrosine	9.30	10.00	10.50	9.10	9.30	9.70
GABA	0.10	0.10	0.10	0.10	0.10	0.10

PC = positive control; NC = negative control; GABA = gamma-aminobutyric acid.

1 PC, high fishmeal diet (200 g/kg); PC400, PC, diet supplemented with protease at 400 mg/kg; PC800, PC, diet supplemented with protease at 800 mg/kg; NC, low fishmeal diet (100 g/kg); NC400, NC, diet supplemented with protease at 400 mg/kg; NC800, NC, diet supplemented with protease at 800 mg/kg.

### 2.3 Experiment design and feeding trial

The feeding trial was conducted at the Marine Science Institute of Jeju National University (Jeju, South Korea). Shrimp were obtained from a hatchery, and fed a commercial diet (40% protein, Woosung premium aqua feed, South Korea) for 3 wk to acclimate them to experimental conditions. After the conditioning, groups of 30 healthy and uniform size juvenile shrimp in intermolt stage (initial weight 0.30 ± 0.01 g) were randomly stocked in 36 aquariums (240 L). Experimental diets were randomly allocated to six replicate groups. Shrimp were fed six times (08:00, 10:00, 12:00, 14:00, 16:00, and 18:00) daily for 8 wk. The biomass in each tank was weighed every 2 wk in order to adjust the feeding rate. The daily feeding ration was initially set at 10% of the total biomass of each aquarium and gradually decreased to 4%, to prevent overfeeding and to ensure apparent satiation. The daily feed amount was divided equally into six portions and fed each turn. If mortalities occurred, the weight of the dead shrimp was recorded, and the feeding rate was adjusted accordingly. The rearing water (about 50%) was renewed every three days with filtered and preheated seawater. A heater to maintain temperature (about 30 °C) and continuous aeration were provided to all tanks. The insides of the tanks were routinely cleaned to prevent microflora growth. During the feeding trial, water quality parameters were monitored daily and maintained within the following ranges: dissolved oxygen 6.12 ± 0.61 mg/L, temperature 30.1 ± 1.4 °C, ammonia 0.04 ± 0.05 mg/L, pH 7.25 ± 0.18 and salinity 32.1 ± 0.3 g/L. At the end of the feeding trial, shrimp were deprived of the feeds for 18 h. Shrimp count and individual weight were recorded in each tank to calculate the following growth metrics: final body weight (FBW), weight gain (WG), specific growth rate (SGR), feed intake (FI), feed conversion ratio (FCR) and protein efficiency ratio (PER).

### 2.4 Sample collection

Six average-sized shrimp were selected from each tank (36 shrimp per treatment) and anesthetized in ice water. Hemolymph was extracted from the ventral sinus of shrimp (three shrimp per tank, *n* = 3) and combined with an anticoagulant (Alsever's solution, Sigma–Aldrich, St. Louis, USA) in a 1:2 ratio. The hemolymph was centrifuged at 800 × *g* at 4 °C for 20 min, and the supernatant was stored at −80 °C to determine immune and antioxidant parameters. Hepatopancreases from six shrimp per tank were frozen in liquid nitrogen and stored at −80 °C for subsequent analysis of gene expression (*n* = 3) and digestive enzyme activity (*n* = 3). Intestine (mid-gut) samples from two shrimp per tank (*n* = 2) were fixed in Davidson's fixative solution for histological analysis. Four shrimp in each tank (pooled as one sample, *n* = 1) were preserved at −20 °C to determine the whole-body amino acid profile and proximate composition.

### 2.5 Analysis

#### 2.5.1 Immune response and antioxidant status

For the immune response analysis, lysozyme activity was measured by the turbidimetric method, as described in Shin et al. (2023). Briefly, a mixture of hemolymph (20 μL) and *Micrococcus lysodeikticus* bacterial suspension (200 μL; 0.75 mg/mL, in 0.1 mol/L sodium phosphate buffer pH 6.4) was incubated at 37 °C and the decrease in optical density was recorded at 570 nm. Phenoloxidase (PO) activity was analyzed by measuring the dopachrome formation as described in Hasanthi and Lee (2023). Hemolymph (50 μL) was treated with trypsin (1 mg/mL in sodium cacodylate buffer) and incubated at 25 °C for 30 min, followed by the addition of L-3,4-dihydroxyphenylalanine (3 mg/mL in sodium cacodylate buffer) and incubation for 30 min. The optical density was then measured at 492 nm. Intracellular production of reactive oxygen species was quantified by nitro-blue tetrazolium (NBT) assay (Zhang et al., 2013). Briefly, hemolymph (40 μL) was mixed with Hank's balanced salt solution (200 μL) and incubated at 25 °C for 30 min, followed by the addition of 100 μL of zymosan and further incubation at 37 °C for 2 h. Subsequently, methanol (600 μL) was added, and after centrifugation, the fixed hemocytes were washed with 70% methanol, then dried and dissolved in a 2 mol/L KOH and dimethyl sulfoxide solution. The absorbance of the resulting mixture was measured at 620 nm. The total hemocyte count (THC) and hyaline cells (HC) were counted with a hemocytometer using an inverted phase-contrast microscope (Leica DMIL, Leica Microsystems GmbH, Wetzlar, Germany). Glutathione peroxidase (GPx), catalase (CAT) and superoxide dismutase (SOD) activities were assessed by using colorimetric assay kits (Biovision Incorporated, CA, USA).

#### 2.5.2 Analysis of proximate composition

The proximate composition was analyzed according to the standard methods (AOAC, 2005). Dry matter content was determined by drying the samples at 105 °C until a constant weight (method 2001.12). Crude protein was quantified by the Kjeldahl method (% nitrogen × 6.25) (method 2001.11). Ash content was determined by incineration of samples at 550 °C for 8 h (method 942.05). The chloroform/methanol extraction method described by Folch et al. (1957) was used to determine the crude lipid. The organic matter content was calculated by subtracting the ash content from the dry matter of sample. Amino acid composition was analyzed by high-performance liquid chromatography (HPLC) equipped with a photodiode array detector (Waters, Milford, MA, USA). Sample preparation and ultrasonic extraction was performed as described in Ding et al. (2023). HPLC analysis was performed using a XB-C18 column (4.6 mm × 250 mm; 5 μm particle) with a mobile phase of acetonitrile:water (4:1) and 0.05 mol/L ammonium acetate (pH 6.5). The injection volume was 5 μL, and the column temperature was 40 °C. The total amount of carbohydrate was calculated on a dry matter basis as follows: 100 – (protein + lipid + ash + fiber). The total phosphorus content was analyzed by the colorimetric method and measured spectrophotometrically (method 995.11). Protease activity of the test diets was determined using the SOY-101/04E method (EFSA FEEDAP Panel, 2023).

#### 2.5.3 Hepatopancreas digestive enzyme analysis

Hepatopancreases were weighed and homogenized with distilled water (1:2, w/v) and subsequently centrifuged at 10,000 × *g* at 4 °C for 15 min to obtain enzyme extracts. The total soluble protein was determined using the BioRad protein assay kit (Cat. No. 5000002, Bio-Rad, Hercules, CA, USA), based on Bradford (1976). Trypsin, pepsin and chymotrypsin activities were determined using established protocols with specific substrates: benzoyl-DL-arginine-p-nitroanilide (BAPNA), hemoglobin (2%, in 0.06 mol/L HCl) and SAPNA, respectively (Erlanger et al., 1961; Worthington, 1993; Falcón-Hidalgo et al., 2011). Enzyme activities were expressed as follows: trypsin activity in BAPNA units/mg protein, pepsin activity in specific activity U and chymotrypsin activity in SAPNA units/mg protein. Amylase activity was measured using 1% starch in 20 mmol/L phosphate buffer containing 6.0 mmol/L NaCl (pH 6.9) as the substrate, following the method of Worthington (1993). The activity was expressed as the released maltose (μmol)/mg protein. Lipase activity was assayed using a stabilized olive oil emulsion, with activity (U) quantified based on the amount of 0.01 mol/L NaOH required to neutralize the released fatty acids during the 6 h incubation, following the method of Borlongan (1990).

#### 2.5.4 Real-time quantitative PCR analysis (RT-qPCR)

In hepatopancreas tissues, total RNA was extracted using Trizol reagent (T9424, Sigma–Aldrich, St. Louis, USA). The purity and concentration of the extracted RNA were measured using a NanoDrop spectrophotometer (Thermo Scientific, USA) at 260 nm. A total of 2.5 μg RNA was reverse transcribed using a cDNA synthesis kit (TaKaRa Code. DRR047, TaKaRa, Shiga, Japan). Gene expression analyses were performed by RT-qPCR (TaKaRa). The primer pairs were designed for β-actin (reference gene; confirmed to be stably expressed in all dietary treatments), *proPO*, crustin, *TGF-β*, *LvIKK-β*, *TLR3* and *TNF-α* based on the published *P. vannamei* cDNA sequence on NCBI GenBank (Table 3). The analysis was programmed as mentioned in Hasanthi et al. (2024): one cycle at 95 °C for 10 s; 45 cycles of 95 °C for 5 s, 60 °C for 20 s; and 72 °C for 20 s; and a final single cycle of 95 °C for 15 s, 60 °C for 30 s and 95 °C for 15 s. The relative mRNA expressions were quantitatively analyzed, and fold changes were obtained according to Pfaffl (2001).

**Table 3:** Sequences of primers used for RT-qPCR.

Target gene	Forward primer sequences (5′ to 3′)	Reverse primer sequences (5′ to 3′)	Accession number/reference
β-Actin	GAGCAACACGGAGTTCGTTGT	CATCACCAACTGGGACGACATGGA	AF300705.2
Crustin	CTTGCACACGTGTTCTCCCAAACA	ACCAAGATACTCGACTGCCCACAA	AY486426.1
*LvIKKβ*	GTACGCACAAAGCAGTCCCATCAT	CATGATGCGCAACCACTCCTCTATTC	JN180642.1
*proPO*	CGCAACGGTGACAAAGTTCCTCTT	TATGTTGTGCAGGTCGCCGTAGTA	AY723296.1
*TGF-β*	GGCCAGTGCCCACAAGAAGAA	CTTCATCTTGCACGCCGTCTCTG	MH259763.1
*TLR3*	TGACGACCTGCATGAACACCTCTT	GGTGCCACGGTACACGAAACATAAC	JN180638.1
*TNF-α*	GGATCGCAGTCAACGCACATGAT	GCAGGGAATAAGGCAGCAACCAA	JN180641.1

*LvIKKβ* = *Litopenaeus vannamei* inhibitor of nuclear factor kappa-β kinase subunit beta; *proPO* = prophenoloxidase; *TGF-β* = transforming growth factor beta; *TLR3* = Toll-like receptor 3; *TNF-α* = tumor necrosis factor α.

### 2.6 Estimation of apparent digestibility coefficients

Experimental diets for the digestibility test were prepared by including 1% chromium (III) oxide (Cr_2_O_3_; Sigma–Aldrich, St. Louis, MO, USA) to the standard diets used in the feeding trial as an inert indicator. After the feeding trial, 60 shrimp (mean weight: approximately 8 g) from each dietary treatment were transferred into triplicate tanks (120 L). Shrimp were fed their respective diets and acclimated for 5 d prior to fecal collection. Shrimp were fed 5% of body weight at 08:30, 12:30, 15:30 and 18:30. About 70% of breeding water was exchanged daily, and uneaten feed and excrement were drained out 30 min post-feeding. Feces (only intact and fresh samples) were collected using a Pasteur pipette three times daily (at 11:00, 14:30 and 17:30) for 3 wk. The feces were separated through filter paper and then frozen at −20 °C until required. Feces samples were pooled within each tank and freeze-dried prior to analysis. The Cr_2_O_3_ content in both feces and diet samples was quantified through acid digestion, following the method described in Divakaran et al. (2002). The apparent digestibility coefficients (ADC) of dry matter (ADCd), lipid (ADCl), protein (ADCp), carbohydrate (ADCc), phosphorus (ADCph) and amino acids (ADCaa) of the diets were calculated by the following equation:

ADCd (%) = 100 – 100 × (%Cr_2_O_3_ in diet/%Cr_2_O_3_ in feces);

ADC of nutrients (%) = 100 – 100 × (%Cr_2_O_3_ in diet/%Cr_2_O_3_ in feces) × (%Nutrient in feces/%Nutrient in diet).

### 2.7 Intestinal histological analysis

The intestine samples were fixed in Davidson's solution for 24 h and then transferred to 70% ethanol. Fixed samples were dehydrated in graded concentrations of ethanol prior to paraffin embedding. Samples were then sectioned to a thickness of 5 μm using a rotary microtome, and the cross-sections were mounted onto microscope slides. The sectioned tissues were stained with Harris hematoxylin and 0.5% eosin and then examined under a microscope for morphological structure. The tissue processing and staining procedure was conducted following the methods outlined by Feldman and Wolfe (2014). The villus length (VL) was measured using the image analyzing software (Leica Application Suite, version 4.13.0, Switzerland).

### 2.8 Gut microbiome analysis

Shrimp gut microbiome was assessed as described by Silva et al. (2013). Two shrimp from each tank were anesthetized with ice and dissected under sterile conditions to obtain gut samples. Each sample was weighed and homogenized with 1 mL of sterile 3% saline solution. The homogenate was centrifuged at 5000 × *g* at 4 °C for 5 min and supernatant was serially diluted 1:10. An aliquot of 100 μL of diluted supernatant was spread on culture mediums. Selective culture mediums were utilized to isolate specific bacteria: marine agar for total heterotrophic marine bacteria, phenylethyl alcohol agar for Gram-positive bacteria, MacConkey agar for Gram-negative bacteria, thiosulfate–citrate–bile salts–sucrose agar for *Vibrio* species and De Man, Rogosa and Sharpe agar (MRS) for lactic acid bacteria. All cultures were incubated at 28 °C for 24 h, except for those on MRS agar, which required 48 h. The total count of the CFU per gram was then enumerated to determine bacterial abundance.

### 2.9 Statistical analysis

Data are presented as mean and standard error of the mean (SEM). A two-way analysis of variance (ANOVA) was performed using the GLM procedure of SAS version 9.3 (SAS Institute, Cary, NC, USA) to determine the main effects of FM and protease and their interaction.

The statistical model is as follows:

*Y*_*ijk*_ = *μ* + *α*_*i*_ + *β*_*j*_ + (*αβ*)_*ij*_ + *ϵ*_*ijk*,_

where *Y*_ijk_ denoted the dependent variable (the *k*^th^ observation on *i*^th^ level of FM and *j*^th^ level of protease), μ denoted the overall mean, *α*_*i*_ denoted the effect of i^th^ level of FM, *β*_j_ denoted the effect of j^th^ level of protease, (*αβ*)_ij_ denoted the interaction effect between *i*^th^ level of FM and *j*^th^ level of protease, and *ϵ*_*ijk*_ denoted the random error. Statistical significance was determined at *P* < 0.05. In case of significant interaction, the differences in treatment means were compared using Tukey's HSD test. Principal component analysis (PCA) was performed using R software (4.2.2) in order to relate dietary FM and protease inclusion levels to amino acid digestibility and exhibit differences among groups.

## 3 Results

### 3.1 Growth performance and feed utilization

Shrimp growth and feed utilization efficiency were significantly (*P* < 0.001) affected by FM and protease levels; however, no significant interaction effect was observed (Table 4). The PC diet significantly (*P* < 0.001) increased the growth and feed utilization efficiency of shrimp compared to the NC diet. The supplementation of protease in either high or low FM diets significantly (*P* < 0.001) improved FBW, SGR, WG, FI and PER, while reducing the FCR. NC400 and NC800 diets significantly increased the FBW, WG, FI and SGR compared to the NC group; however, they did not reach levels comparable to the PC group. The inclusion of protease in LFM diet decreased FCR and increased PER, achieving levels similar to those in PC group. Protease, FM level and their interaction showed no significant effect (*P* > 0.05) on shrimp survival.

**Table 4:** Growth performance, feed utilization and survival of Pacific white shrimp (*Penaeus vannamei*) fed the experimental diets for 8 wk.

Item	Experimental diets^1^							Two-way ANOVA: *P* values		
	PC	PC400	PC800	NC	NC400	NC800	SEM	FM replacement level	Protease level	FM × Protease
FBW, g	8.89^b^	9.44^a^	9.73^a^	7.43^d^	8.14^c^	8.25^c^	0.086	<0.001	<0.001	0.554
WG^2^, %	2861.27^b^	3052.17^a^	3122.35^a^	2377.37^d^	2597.99^c^	2637.04^c^	33.012	<0.001	<0.001	0.868
SGR^3^, %/d	6.16^b^	6.27^a^	6.31^a^	5.84^d^	5.99^c^	6.02^c^	0.021	<0.001	<0.001	0.601
FI^4^, g/shrimp	10.51^b^	10.74^a^	10.85^a^	9.15^e^	9.71^d^	9.89^c^	0.962	<0.001	<0.001	<0.001
FCR^5^	1.21^bc^	1.16^cd^	1.14^d^	1.27^a^	1.22^ab^	1.23^ab^	0.042	<0.001	<0.001	0.280
PER^6^	1.99^bc^	2.08^ab^	2.12^a^	1.89^d^	1.97^cd^	1.95^cd^	0.013	<0.001	<0.001	0.271
SR^7^, %	96.67	96.11	96.67	97.78	96.67	97.22	0.021	0.353	0.681	0.946

PC = positive control; NC = negative control; FM = fishmeal; FBW = final mean body weight; WG = weight gain; SGR = specific growth rate; FI = feed intake; FCR = feed conversion ratio; PER = protein efficiency ratio; SR = survival.

Values are mean of six replicate groups and presented as means and SEM. Values with different superscripts in the same row are significantly different (*P* < 0.05).

1 PC, high FM, diet (200 g/kg); PC400, PC, diet supplemented with protease at 400 mg/kg; PC800, PC, diet supplemented with protease at 800 mg/kg; NC, low FM, diet (100 g/kg); NC400, NC, diet supplemented with protease at 400 mg/kg; NC800, NC, diet supplemented with protease at 800 mg/kg.

2 WG = [(Final body weight – Initial body weight)/Initial body weight] × 100.

3 SGR = [(Ln final body weight – Ln initial body weight)/days] × 100.

4 FI = (Dry feed given – Dry remaining feed recovered)/Number of shrimp.

5 FCR = Dry feed intake (g)/Wet weight gain (g).

6 PER = wet weight gain (g)/total protein given (g).

7 SR = (Final shrimp number/Initial shrimp number) × 100.

### 3.2 Non-specific immune responses and antioxidant capacity

Non-specific immune responses and antioxidant enzyme activities were significantly affected (*P* < 0.001) by FM and protease levels; however, their interaction effects showed no significant influence (*P >* 0.05), except for GPx (*P* = 0.048) (Table 5). Shrimp fed the PC diet exhibited significantly higher (*P* < 0.001) lysozyme, PO, NBT, THC, HC, SOD and CAT activities compared to those fed the NC diet. Notably, supplementation of protease in LFM diet enhanced PO, lysozyme, NBT, THC, SOD and GPx activities (*P* < 0.001) to levels comparable to the PC group.

**Table 5:** Non-specific immune parameters and antioxidant enzyme activities of Pacific white shrimp (*Penaeus vannamei*) fed the experimental diets for 8 wk.

Item	Experimental diets^1^							Two-way ANOVA: *P* values		
	PC	PC400	PC800	NC	NC400	NC800	SEM	FM replacement level	Protease level	FM × Protease
PO, ΔOD_492_/min per mL	0.20^b^	0.20^b^	0.23^a^	0.17^c^	0.19^bc^	0.19^b^	0.006	<0.001	<0.001	0.219
Lyz, μg/mL	4.01^bc^	4.47^ab^	4.92^a^	2.80^d^	3.65^c^	3.77^bc^	0.171	<0.001	<0.001	0.463
NBT, ΔOD_620_/min per mL	3.12^b^	3.28^ab^	3.48^a^	2.65^c^	3.06^b^	3.09^b^	0.065	<0.001	<0.001	0.184
THC, × 10^5^ cells/mL	276.67^ab^	285.28^a^	289.72^a^	254.44^c^	264.44^bc^	270.56^b^	3.186	<0.001	<0.001	0.891
HC, × 10^5^ cells/mL	79.44^bc^	84.44^ab^	89.72^a^	68.33^e^	69.72^de^	75.83^cd^	1.628	<0.001	<0.001	0.517
SOD^2^	45.76^abc^	49.44^ab^	52.13^a^	32.68^d^	41.96^c^	44.79^bc^	1.592	<0.001	<0.001	0.139
GPx, mU/mL	68.64^ab^	81.76^a^	78.66^ab^	46.95^d^	53.57^cd^	66.64^bc^	3.142	<0.001	<0.001	0.048
CAT, mU/mL	2.68^b^	2.92^b^	3.40^a^	2.13^d^	2.27^cd^	2.60^bc^	0.090	<0.001	<0.001	0.379

PC = positive control; NC = negative control; FM = fishmeal; PO = phenoloxidase; OD = optical density; Lyz = lysozyme; NBT = nitro blue tetrazolium; THC = total haemocyte count; HC = hyaline cell count; SOD = superoxide dismutase; GPx = glutathione peroxidase; CAT = catalase.

Values are mean of six replicate groups and presented as means and SEM. Values with different superscripts in the same row are significantly different (*P* < 0.05).

1 PC, high FM, diet (200 g/kg); PC400, PC, diet supplemented with protease at 400 mg/kg; PC800, PC, diet supplemented with protease at 800 mg/kg; NC, low FM, diet (100 g/kg); NC400, NC, diet supplemented with protease at 400 mg/kg; NC800, NC, diet supplemented with protease at 800 mg/kg.

2 Superoxide dismutase activity was expressed as the percentage inhibition rate using a commercial assay kit (Biovision Incorporated, CA, USA), following the manufacturer's protocol.

### 3.3 Gene expression

**Fig. 1:**
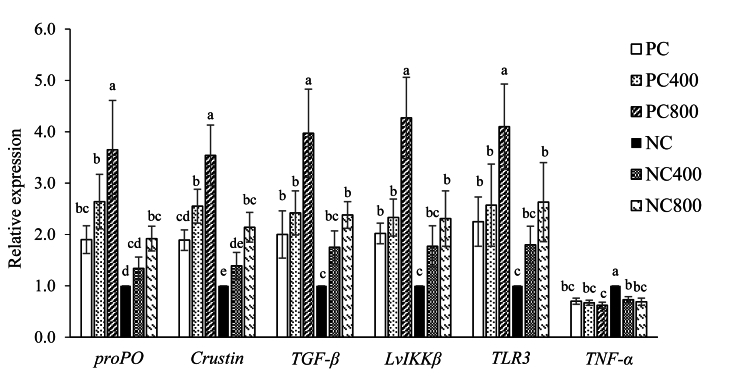
The relative gene expression level in Pacific white shrimp (*Penaeus vannamei*) fed the experimental diets for 8 wk. Values are mean of six replicate groups and presented as mean ± SEM. The different letters on the bars of the same gene indicate significant differences (*P* < 0.05) among treatments. PC, high FM diet (200 g/kg); PC400, PC diet supplemented with protease at 400 mg/kg; PC800, PC diet supplemented with protease at 800 mg/kg; NC, low FM diet (100 g/kg); NC400, NC diet supplemented with protease at 400 mg/kg; NC800, NC diet supplemented with protease at 800 mg/kg. FM = fishmeal; *proPO* = prophenoloxidase; *TGF-β* = transforming growth factor beta; *LvIKKβ* = inhibitor of nuclear factor kappa-β kinase subunit beta; *TLR3* = toll-like receptor 3; *TNF-α* = tumor necrosis factor α.

FM and protease levels significantly affected (*P* < 0.001) the expression of *proPO*, crustin, *TGF-β*, *LvIKK-β*, *TLR3* and *TNF-α*, while their interaction significantly influenced *LvIKK-β* (*P* = 0.003) and *TNF-α* (*P* < 0.001) genes (Fig. 1). Increasing FM and protease levels significantly upregulated the *proPO*, crustin, *TGF-β*, *LvIKK-β* and *TLR3* genes (*P* < 0.001), with the highest expression observed in the PC800 group, whereas the lowest was exhibited in the NC group. In contrast, *TNF-α* showed the opposite correlation. The supplementation of exogenous protease in LFM diets upregulated (*P* < 0.001) the expression of all aforementioned immune and inflammation-related genes to levels comparable to the PC group.

### 3.4 Digestive enzyme activity

Trypsin, pepsin and lipase activities were significantly affected (*P* < 0.001) by FM and protease levels, while their interaction also affected on pepsin activity (*P* = 0.035) (Table 6). Chymotrypsin (*P* = 0.002) and amylase (*P* = 0.009) activities were only affected by FM level. Supplementation of protease in LFM diet improved the digestive enzyme activity, resulting similar levels (*P* > 0.05) of trypsin, chymotrypsin and pepsin activities among NC400, NC800, PC and PC400 groups. Lipase activity exhibited comparable levels between NC800 and PC groups (*P* = 0.993). Amylase activity exerted no significant difference (*P* > 0.05) between dietary treatments.

**Table 6:** Digestive enzyme activities in hepatopancreas of Pacific white shrimp (*Penaeus vannamei*) fed experimental diets for 8 wk.

Item	Experimental diets^1^							Two-way ANOVA: *P* values		
	PC	PC400	PC800	NC	NC400	NC800	SEM	FM replacement level	Protease level	FM × Protease
Trypsin, BAPNA unit/mg protein	20.04^ab^	20.51^ab^	22.82^a^	16.22^c^	18.63^bc^	18.61^bc^	0.760	<0.001	0.007	0.275
Chymotrypsin, SAPNA unit/mg protein	7.31^a^	7.27^a^	7.45^a^	6.44^b^	6.94^ab^	7.07^ab^	0.188	0.002	0.136	0.310
Pepsin, U/mg protein	1.32^b^	1.31^b^	1.73^a^	0.98^c^	1.19^bc^	1.24^bc^	0.069	<0.001	<0.001	0.035
Amylase, U/mg protein	1.75	1.77	1.80	1.61	1.65	1.68	0.090	0.009	0.569	0.980
Lipase, U/mg protein	2.11^bc^	2.24^ab^	2.32^a^	1.80^e^	1.91^de^	2.00^cd^	0.045	<0.001	<0.001	0.993

PC, positive control; NC, negative control; FM, fishmeal; BAPNA, benzoyl-DL-arginine-p-nitroanilide; SAPNA = N-succinyl-Ala-Ala-Pro-Phe-p-nitroanilide.

Values are mean of six replicate groups and presented as means and SEM. Values with different superscripts in the same row are significantly different (*P* < 0.05).

1 PC, high FM, diet (200 g/kg); PC400, PC, diet supplemented with protease at 400 mg/kg; PC800, PC, diet supplemented with protease at 800 mg/kg; NC, low FM, diet (100 g/kg); NC400, NC, diet supplemented with protease at 400 mg/kg; NC800, NC, diet supplemented with protease at 800 mg/kg.

### 3.5 Apparent digestibility coefficient of nutrients

ADCd, ADCl, ADCp and ADCph were significantly improved (*P* < 0.001) by FM and protease levels, while their interaction also influenced ADCd (*P* = 0.030) and ADCp (*P* = 0.006) (Table 7). ADCc was affected by FM level (*P* < 0.001). The protease supplementation in the LFM diet significantly enhanced ADCl (*P* < 0.001), ADCc (*P* = 0.091) and ADCph (*P* = 0.002) to the levels comparable (*P >* 0.05) to PC group. Notably, ADCd, ADCp and ADCph exhibited no significant difference (*P* > 0.05) between NC400, NC800, PC and PC400 groups.

**Table 7:** Apparent digestibility coefficients (%) of nutrients for the six experimental diets for Pacific white shrimp (*Penaeus vannamei*).

Item	Experimental diets^1^							Two-way ANOVA: *P* values		
	PC	PC400	PC800	NC	NC400	NC800	SEM	FM replacement level	Protease level	FM × Protease
ADCd	66.76^b^	69.71^ab^	72.78^a^	61.37^c^	67.95^b^	66.26^b^	0.570	<0.001	<0.001	0.030
ADCl	69.43^bc^	72.75^ab^	75.60^a^	59.74^d^	67.04^c^	69.67^bc^	0.836	<0.001	<0.001	0.208
ADCp	86.61^c^	88.24^b^	89.88^a^	84.34^d^	87.46^bc^	87.42^bc^	0.162	<0.001	<0.001	0.006
ADCc	84.64^ab^	85.58^a^	85.63^a^	77.80^c^	80.69^bc^	80.91^bc^	0.668	<0.001	0.091	0.482
ADCph	68.49^ab^	70.89^a^	72.40^a^	65.39^b^	68.82^ab^	68.95^ab^	0.608	0.001	0.002	0.713

PC = positive control; NC = negative control; FM, fishmeal; ADC = apparent digestibility coefficients; ADCd = ADC of dry matter; ADCp = ADC of protein; ADCc = ADC of carbohydrate; ADCph = ADC of phosphorus.

Values are mean of triplicate groups and presented as means and SEM. Values with different superscripts in the same row are significantly different (*P* < 0.05).

1 PC, high FM, diet (200 g/kg); PC400, PC, diet supplemented with protease at 400 mg/kg; PC800, PC, diet supplemented with protease at 800 mg/kg; NC, low FM, diet (100 g/kg); NC400, NC, diet supplemented with protease at 400 mg/kg; NC800, NC, diet supplemented with protease at 800 mg/kg.

**Fig. 2:**
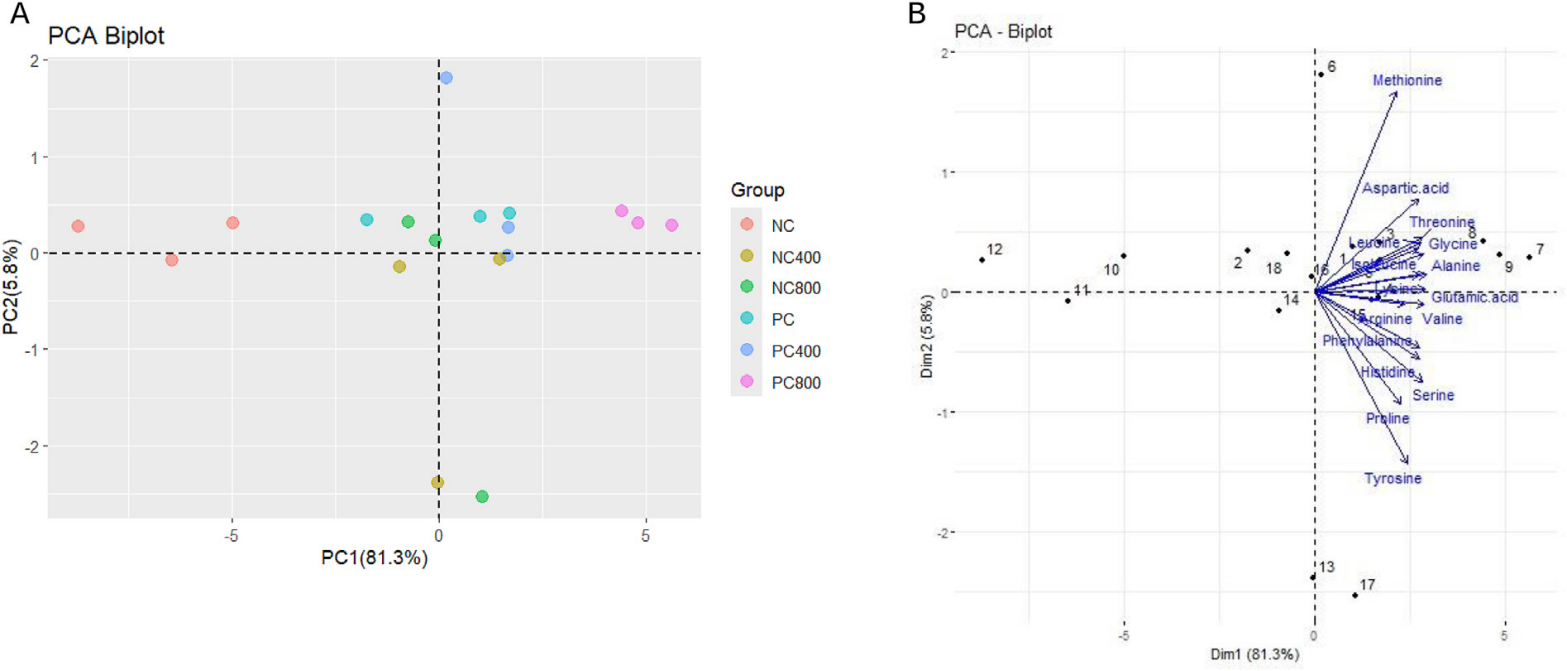
Principal component analysis (PCA) of apparent digestibility coefficients (%, ADC) for amino acids of six experimental diets (NC, NC400, NC800, PC, PC400 and PC800) for Pacific white shrimp (*Penaeus vannamei*). (A) PCA biplot diagram for the first and second principal components, showing the relationship of amino acid digestibility among dietary groups. (B) PCA biplot with amino acids overlaid onto the dietary groups. PC, high fishmeal [FM] diet (200 g/kg); PC400, PC diet supplemented with protease at 400 mg/kg; PC800, PC diet supplemented with protease at 800 mg/kg; NC, low FM diet (100 g/kg); NC400, NC diet supplemented with protease at 400 mg/kg; NC800, NC diet supplemented with protease at 800 mg/kg.

Exogenous protease supplementation in either high or low FM diets enhanced essential and non-essential amino acid digestibility (Table 8). The ADCaa was improved (*P* < 0.05) by increasing FM and protease levels. Protease supplementation in the LFM diet significantly increased amino acids digestibility (*P* < 0.05), resulting in comparable ADCaa among PC, PC400, NC400 and NC800 groups. The PCA analysis exhibited that the first two components represented 87.1% of total variance of amino acid digestibility in the shrimp (Fig. 2). In PCA Biplot (A), PC1 explained 81.3% and PC2 explained 5.8% of the total variance. The separation between dietary treatments could be demonstrated from the graph, where NC400, NC800, PC and PC400 groups were found to be clustered together and close to each other indicating these treatments have similar amino acid digestibility profiles. NC and PC800 groups were found to be separated from other groups exhibiting distinct differences in amino acid digestibility. The PC800 was characterized as the group with highest amino acid digestibility having positive correlation with PC1 along with high magnitude for ADC of glycine, alanine, threonine, glutamic acid and aspartic acid, demonstrating that PC800 has higher digestibility for these specific amino acids. NC, which is located on the opposite side of the plot along PC1, shows a weaker association with these amino acids, indicating lower digestibility.

**Table 8:** Apparent digestibility coefficients (%, ADC) for the amino acid composition of the six experimental diets for Pacific white shrimp (*Penaeus vannamei*).

Item	Experimental diets^1^							Two-way ANOVA: *P* values		
	PC	PC400	PC800	NC	NC400	NC800	SEM	FM replacement level	Protease level	FM × Protease
**Essential amino acids**										
Arginine	81.83^ab^	82.81^a^	84.42^a^	77.11^b^	82.19^ab^	82.61^a^	0.795	0.023	0.010	0.213
Threonine	86.41^b^	84.91^b^	87.01^a^	81.19^c^	83.93^b^	83.17^b^	0.289	<0.001	<0.001	0.009
Valine	86.63^ab^	87.67^ab^	88.59^a^	82.40^c^	86.38^b^	86.27^b^	0.303	<0.001	<0.001	0.015
Phenylalanine	88.19^ab^	88.27^ab^	89.88^a^	85.82^b^	88.07^ab^	87.70^ab^	0.417	0.007	0.030	0.168
Isoleucine	89.26^ab^	90.07^a^	90.84^a^	86.99^b^	89.29^ab^	88.97^ab^	0.356	0.002	0.008	0.347
Leucine	88.73^a^	89.35^a^	90.27^a^	85.59^b^	87.89^ab^	88.10^a^	0.361	<0.001	0.005	0.291
Histidine	89.89^a^	89.49^a^	91.06^a^	87.00^b^	89.97^a^	89.64^a^	0.311	0.004	0.003	0.008
Lysine	91.99^a^	91.84^a^	94.16^a^	89.07^b^	91.98^a^	91.77^a^	0.362	0.001	0.002	0.024
Methionine	90.41^ab^	90.89^ab^	91.98^a^	87.85^b^	89.40^ab^	90.10^ab^	0.493	0.005	0.054	0.745
**Non-essential amino acids**										
Aspartic acid	88.19^ab^	89.16^ab^	90.20^a^	86.13^c^	88.00^bc^	88.12^bc^	0.301	<0.001	0.002	0.480
Alanine	83.74^ab^	83.46^b^	85.93^a^	79.61^c^	82.92^b^	83.06^b^	0.347	<0.001	<0.001	0.010
Serine	86.00^a^	86.44^a^	88.04^a^	82.48^b^	86.29^a^	86.81^a^	0.406	0.005	<0.001	0.034
Glutamic acid	91.97^b^	91.96^b^	93.14^a^	89.90^c^	91.88^b^	91.64^b^	0.172	<0.001	<0.001	0.004
Proline	88.69^ab^	90.65^ab^	94.11^a^	85.41^b^	90.88^ab^	91.93^a^	0.822	0.092	0.001	0.339
Glycine	81.59^b^	82.21^b^	85.40^a^	76.98^c^	81.65^b^	81.70^b^	0.456	<0.001	<0.001	0.021
Tyrosine	87.74^ab^	87.71^ab^	89.39^a^	85.59^b^	89.03^a^	88.83^a^	0.429	0.365	0.005	0.044

PC = positive control; NC = negative control; FM = fishmeal.

Values are mean of triplicate groups and presented as means and SEM. Values with different superscripts in the same row are significantly different (*P* < 0.05).

1 PC, high FM, diet (200 g/kg); PC400, PC, diet supplemented with protease at 400 mg/kg; PC800, PC, diet supplemented with protease at 800 mg/kg; NC, low FM, diet (100 g/kg); NC400, NC, diet supplemented with protease at 400 mg/kg; NC800, NC, diet supplemented with protease at 800 mg/kg.

### 3.6 Intestinal morphology

**Fig. 3:**
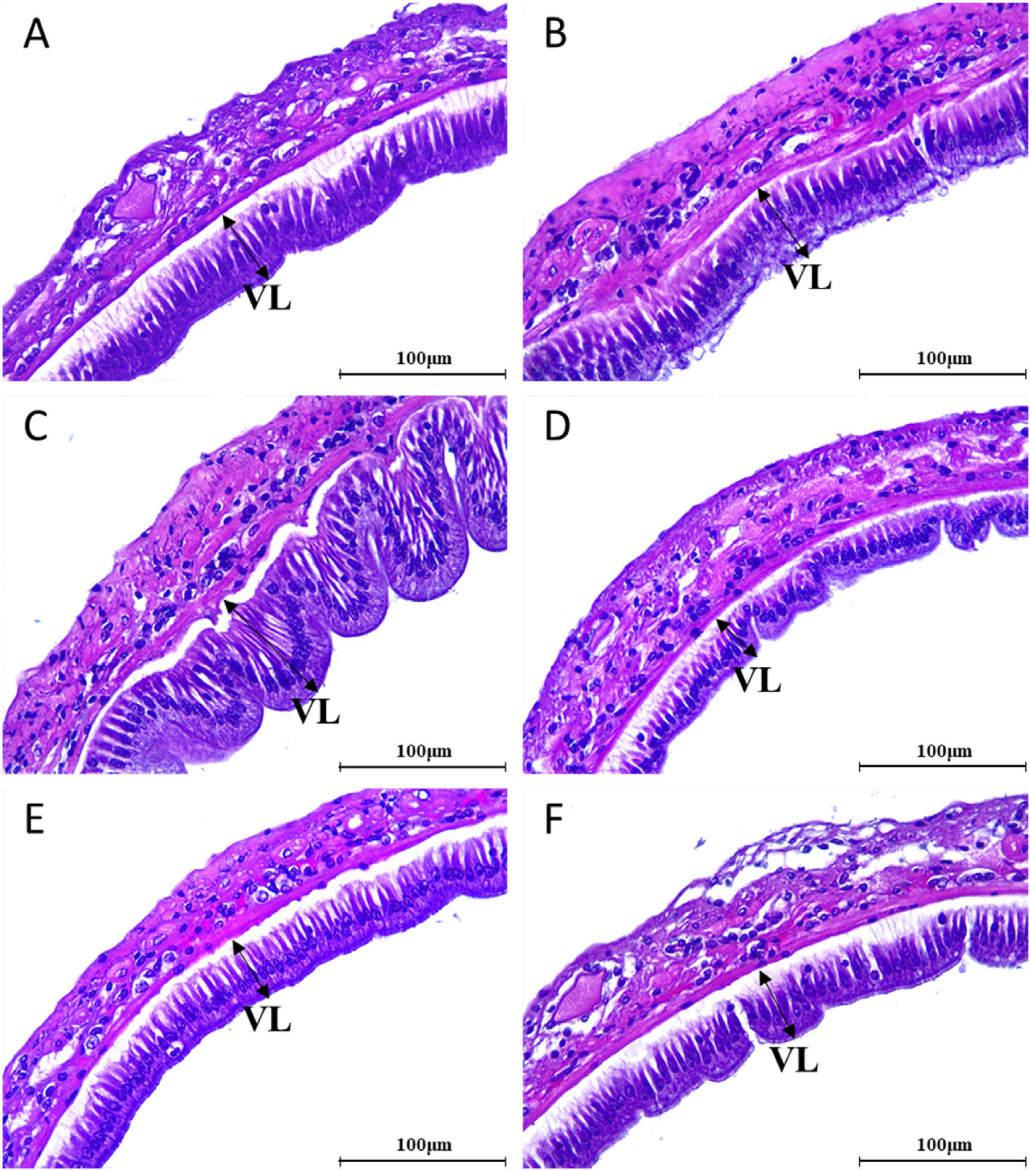
Intestinal morphology of Pacific white shrimp (*Penaeus vannamei*) fed six experimental diets for 8 wk (magnification 40× ). (A) PC, high fishmeal [FM] diet (200 g/kg); (B) PC400, PC diet supplemented with protease at 400 mg/kg; (C) PC800, PC diet supplemented with protease at 800 mg/kg; (D) NC, low FM diet (100 g/kg); (E) NC400, NC diet supplemented with protease at 400 mg/kg; (F) NC800, NC diet supplemented with protease at 800 mg/kg. VL = villus length; PC = positive control; NC = negative control; FM = fishmeal.

Intestinal villus length was significantly affected by FM and protease levels (Table 9). Villi length was significantly increased with increasing FM and protease levels (*P* < 0.001). The highest villi length was observed in PC800 group, while the NC group exhibited the lowest level. Notably, increasing dietary protease level in LFM diet significantly increased intestinal villi length, resulting in comparable levels between NC800 and PC groups. Representative histology images of intestinal cross-sections are shown in Fig. 3.

**Table 9:** Intestinal villus length of Pacific white shrimp (*Penaeus vannamei*) fed six experimental diets for 8 wk^1^.

Item	Villus length, μm
PC	43.07^c^
PC400	45.35^b^
PC800	50.21^a^
NC	34.75^e^
NC400	38.90^d^
NC800	41.49^c^
SEM	0.525
**Two-way ANOVA: *P* values**	
FM replacement level	<0.001
Protease level	<0.001
FM × protease	0.087

PC, positive control; NC, negative control; FM, fishmeal.

Values are mean of six replicate groups and presented as means and SEM, of six replicate groups. Values with different superscripts in the same column are significantly different (*P* < 0.05).

1 PC, high FM, diet (200 g/kg); PC400, PC, diet supplemented with protease at 400 mg/kg; PC800, PC, diet supplemented with protease at 800 mg/kg; NC, low FM, diet (100 g/kg); NC400, NC, diet supplemented with protease at 400 mg/kg; NC800, NC, diet supplemented with protease at 800 mg/kg.

### 3.7 Gut microbial structure

The shrimp gut microbiota was significantly affected by the FM, protease levels and their interaction (Table 10). The heterotrophic bacteria and *Lactobacillus* spp. were significantly increased with higher dietary FM and protease levels (*P* < 0.001), with the highest levels observed in the PC800 group and the lowest in the NC group. Notably, increasing protease level in the LFM diet significantly decreased *Vibrio* spp. (*P* = 0.001), resulting in no significant difference among NC400, PC and PC400 groups. A significantly higher abundance of Gram-positive bacteria was observed in the PC400 group than in the NC group. Neither dietary FM level, protease level, nor their interaction significantly affected Gram-negative bacteria (*P >* 0.05).

**Table 10:** Gut microbiome analysis of Pacific white shrimp (*Penaeus vannamei*) fed the experimental diets for 8 wk.

Item	Experimental diets^1^							Two-way ANOVA: *P* values		
	PC	PC400	PC800	NC	NC400	NC800	SEM	FM replacement level	Protease level	FM × Protease
Heterotrophic marine bacteria, × 10^6^ CFU/g	6.09^c^	13.95^b^	19.70^a^	1.57^d^	4.25^cd^	5.14^cd^	0.927	<0.001	<0.001	<0.001
*Vibrio* spp., × 10^3^ CFU/g	5.12^c^	4.37^cd^	2.86^d^	9.17^a^	6.40^bc^	7.84^ab^	0.494	<0.001	0.001	0.014
Gram negative bacteria, × 10^3^ CFU/g	1.78	2.05	1.52	2.19	1.92	1.95	0.234	0.218	0.480	0.416
Gram positive bacteria, × 10^4^ CFU/g	3.77^ab^	5.90^a^	4.24^ab^	3.44^b^	3.59^b^	3.79^ab^	0.468	0.005	0.034	0.044
*Lactobacilli* spp., × 10^2^ CFU/g	4.96^bc^	5.69^b^	9.24^a^	2.32^c^	3.58^bc^	4.63^bc^	0.713	<0.001	<0.001	0.146

PC, positive control; NC, negative control; FM, fishmeal.

Values are mean of six replicate groups and presented as means and SEM., Values with different superscripts in the same row are significantly different (*P* < 0.05).

1 PC, high FM, diet (200 g/kg); PC400, PC, diet supplemented with protease at 400 mg/kg; PC800, PC, diet supplemented with protease at 800 mg/kg; NC, low FM, diet (100 g/kg); NC400, NC, diet supplemented with protease at 400 mg/kg; NC800, NC, diet supplemented with protease at 800 mg/kg.

### 3.8 Whole-body amino acid and proximate composition

Increasing dietary FM and protease levels significantly increased whole-body essential and non-essential amino acid content (*P* < 0.05) (Table 11). A significantly higher whole-body amino acid profile was observed in NC400 and NC800 groups than in NC group for arginine (*P* = 0.013), threonine (*P* = 0.007), phenylalanine (*P* = 0.022), lysine (*P* = 0.026), taurine (*P* = 0.036) and glutamic acid (*P* = 0.041), while comparable levels were observed between PC, PC400, NC400 and NC800 groups.

**Table 11:** The whole-body amino acid composition (g/100 g, DM basis) of Pacific white shrimp (*Penaeus vannamei*).

Item	Experimental diets^1^							Two-way ANOVA: *P* values		
	PC	PC400	PC800	NC	NC400	NC800	SEM	FM replacement level	Protease level	FM × Protease
**Essential amino acids**										
Arginine	7.70^ab^	7.75^a^	7.76^a^	7.55^b^	7.66^ab^	7.76^a^	0.018	0.028	0.013	0.116
Threonine	2.72^ab^	2.72^ab^	2.78^a^	2.66^b^	2.66^b^	2.74^ab^	0.010	0.012	0.007	0.751
Valine	3.05^ab^	3.08^ab^	3.16^a^	2.80^b^	2.94^ab^	3.02^ab^	0.034	0.011	0.099	0.571
Phenylalanine	2.92^ab^	2.94^ab^	3.06^a^	2.83^b^	2.84^b^	2.89^b^	0.016	0.001	0.022	0.302
Isoleucine	2.68	2.69	2.73	2.58	2.61	2.65	0.017	0.010	0.171	0.966
Leucine	4.65^ab^	4.66^ab^	4.74^a^	4.50^b^	4.53^b^	4.61^ab^	0.021	0.004	0.068	0.915
Histidine	1.60	1.63	1.65	1.52	1.52	1.59	0.017	0.016	0.207	0.747
Lysine	4.18^ab^	4.21^ab^	4.26^a^	3.97^b^	4.17^ab^	4.22^a^	0.025	0.046	0.026	0.157
Methionine	1.40^ab^	1.44^ab^	1.50^a^	1.32^b^	1.39^ab^	1.40^ab^	0.016	0.019	0.051	0.772
Taurine	0.52^ab^	0.52^a^	0.54^a^	0.47^b^	0.50^ab^	0.51^ab^	0.005	0.003	0.036	0.405
**Non-essential amino acids**										
Aspartic acid	6.62	6.76	6.79	6.34	6.49	6.58	0.053	0.016	0.150	0.907
Alanine	4.32^ab^	4.34^ab^	4.49^a^	4.15^b^	4.21^ab^	4.31^ab^	0.032	0.014	0.066	0.910
Serine	2.68	2.60	2.68	2.61	2.58	2.61	0.027	0.211	0.463	0.867
Glutamic acid	11.17^ab^	11.04^ab^	11.63^a^	10.77^b^	10.76^b^	10.97^ab^	0.072	0.005	0.041	0.373
Proline	2.50^ab^	2.51^ab^	2.56^a^	2.25^b^	2.32^ab^	2.46^ab^	0.030	0.005	0.085	0.402
Glycine	5.25	5.26	5.42	5.30	5.37	5.40	0.075	0.722	0.600	0.903
Tyrosine	2.37	2.39	2.40	2.27	2.29	2.36	0.029	0.095	0.542	0.782

PC, positive control; NC, negative control; FM, fishmeal.

Values are mean of six replicate groups and presented as means and SEM., Values with different superscripts in the same row are significantly different (*P* < 0.05).

1 PC, high FM, diet (200 g/kg); PC400, PC, diet supplemented with protease at 400 mg/kg; PC800, PC, diet supplemented with protease at 800 mg/kg; NC, low FM, diet (100 g/kg); NC400, NC, diet supplemented with protease at 400 mg/kg; NC800, NC, diet supplemented with protease at 800 mg/kg.

Table 12 illustrates the whole-body proximate composition. FM level, protease level, or their interaction did not significantly affect (*P* > 0.05) the dry matter, lipid, protein and ash content.

**Table 12:** Whole-body proximate composition (%, wet weight basis) of Pacific white shrimp (*Penaeus vannamei*) fed the experimental diets for 8 wk.

Item	Experimental diets^1^							Two-way ANOVA: *P* values		
	PC	PC400	PC800	NC	NC400	NC800	SEM	FM replacement level	Protease level	FM × Protease
Dry matter	23.25	23.78	23.50	22.82	23.22	23.72	0.247	0.062	0.171	0.084
Protein	17.52	18.01	18.15	17.46	17.67	17.83	0.203	0.157	0.055	0.741
Lipid	1.07	1.07	1.08	1.04	1.04	1.06	0.051	0.551	0.937	0.974
Ash	3.79	3.71	3.78	3.85	3.76	3.86	0.149	0.609	0.822	0.991

PC = positive control; NC = negative control; FM = fishmeal.

Values are mean of six replicate groups and presented as means and SEM.

1 PC, high FM, diet (200 g/kg); PC400, PC, diet supplemented with protease at 400 mg/kg; PC800, PC, diet supplemented with protease at 800 mg/kg; NC, low FM, diet (100 g/kg); NC400, NC, diet supplemented with protease at 400 mg/kg; NC800, NC, diet supplemented with protease at 800 mg/kg.

## 4 Discussion

In the present study, shrimp fed the HFM diet (200 g/kg) exhibited higher growth and better feed utilization compared to those fed the LFM diet (100 g/kg). Fishmeal is considered an ideal protein source for aquafeed production due to its well-balanced amino acid profile, essential fatty acids, phospholipids, nucleotides, vitamins and minerals, and it can enhance palatability and nutrient digestibility (Mugwanya et al., 2023). In contrast, soybean meal contains various ANFs such as phytic acid, saponins, protease inhibitors, glycinin, lectins, oligosaccharides and β-conglycinin, which interfere with nutrient digestibility and bioavailability (Padalkar et al., 2023). Additionally, soybean meal is deficient in several essential amino acids, with methionine being the most limiting, followed by lysine (Nunes et al., 2014). This could contribute to the negative physiological impacts observed in shrimp fed the NC diet compared to those fed the PC diet. Thus, an efficient formulation strategy is essential to maximize the effectiveness of LFM diets in meeting the nutritional requirements of fish and shrimp species.

In the present study, exogenous protease enhanced growth, nutrient digestibility, feed utilization efficiency and intestinal villi length in shrimp fed either HFM or LFM diets. Notably, the shrimp fed the protease-supplemented LFM diet performed similarly to the HFM group. Proteases catalyze proteolytic and hydrolytic reactions, breaking down protein molecules into peptides and free amino acids (Hassaan et al., 2019). They also regulate enzymatic cascades that are part of various metabolic pathways (i.e., lipid and carbohydrate metabolism) by converting inactive proenzyme zymogens into active enzymes (Ramos and Malcata, 2011). Soybean meal, a common ingredient in plant-based diets, contains a wide variety of ANFs that impair fish/shrimp growth performance. Exogenous proteases degrade protease and trypsin inhibitors by disrupting complex protein layers in plant cell walls, thereby facilitating the digestion of indigestible components (Hassaan et al., 2019; Hussain et al., 2019). In the present study, the improved growth and feed utilization efficiency achieved with protease supplementation may result from accelerated protein hydrolysis, increased bioavailability of nutrients through the degradation of ANFs and enhanced nutrient absorption facilitated by improved intestinal structure. Similarly, previous studies demonstrated that exogenous proteases could eliminate the adverse effects of ANFs, improve dietary amino acid profiles and enhance energy utilization, leading to increased growth in rainbow trout (*Oncorhynchus mykiss*) (Farhangi and Carter, 2007) and tilapia (*Oreochromis niloticus* × *O. aureus*) (Lin et al., 2007).

Our findings demonstrated that exogenous protease supplementation enhanced nutrient digestibility. Consistent with our results, previous studies have reported that protease supplementation in plant protein-based diets can improve nutrient digestibility and feed efficiency in various aquatic species (Dalsgaard et al., 2012; Ginindza et al., 2016; Hassaan et al., 2019; Hlophe-Shi et al., 2016). These findings support the role of protease in improving digestion and utilization of enzyme-digested nutrients, thereby enhancing feed efficiency and growth.

In this study, exogenous protease supplementation enhanced hepatopancreas trypsin, chymotrypsin, pepsin and lipase activities. The findings are consistent with previous studies demonstrating that exogenous protease accelerates endogenous digestive enzyme activity in aquatic species (Hassaan et al., 2020; Shi et al., 2016; Song et al., 2017; Zhang et al., 2012). Proteases enhance nutrient digestion, consequently increasing digested nutrients in the gut and stimulating the release of regulatory hormones such as cholecystokinin (CCK)-like peptides (Liddle, 1995; Beglinger and Degen, 2004). CCK, in turn, stimulates the secretion of digestive enzymes, boosting endogenous lipase and protease production (Hildebrand et al., 1998). The dietary addition of protease increased lipase activity in Nile tilapia (Hassaan et al., 2020) and gibel carp (*Carassius auratus gibelio*) (Xu et al., 2022), as well as pancreatic lipase activity and lipase mRNA expression in broilers (Yuan et al., 2017). However, dietary supplementation of exogenous enzymes at high levels was reported to interfere with the structural development of digestive organs and metabolism, which might negatively affect digestive enzyme activity and gastrointestinal function (Yuan et al., 2015).

In the present study, exogenous protease supplementation enhanced non-specific immune responses and antioxidant enzyme activities, accompanied by the modulation of the expression of genes associated with immune functions and inflammation. Specifically, the mRNA expression of *proPO*, crustin, *TGF-β*, *LvIKK-β* and *TLR3* genes was upregulated, while *TNF-α* was downregulated. In crustaceans, the immune defense mechanism mainly depends on innate immunity, which is characterized by cellular and humoral immune responses (Huang et al., 2020). The immune and metabolic systems are intimately linked, as energy and metabolic substrates derived from nutrients serve as precursors for the biosynthesis of protective molecules such as cytokines, antibodies and glutathione (Moura et al., 2017). Proteases, acting as signaling molecules via protease-activated receptors, influence leukocyte motility, macrophage and monocyte proliferation, cytokine production and various pathophysiological functions (Shpacovitch et al., 2008). In invertebrates, the proteolytic cascade, involving different proteases that activate one another, plays a significant role in innate immunity. By activating pattern recognition proteins (PRPs), proteolytic cascades regulate various immunological responses such as melanization, hemolymph coagulation, complement-like reactions and Toll receptor activation (Cerenius et al., 2010). The crustacean clotting mechanism preliminary consists of transglutaminases released upon wounding and plasma clotting proteins activated by PRPs (Theopold et al., 2004). Serine proteinases and pattern recognition factors facilitate the proteolysis of *proPO* and convert it into active PO, which regulates melanization. Wu et al. (2020) reported that exogenous protease increased total antioxidant capacity, CAT, SOD and GPx activities in the intestine of tilapia suggesting that optimal dietary protease supplementation could reduce intestinal oxidative stress in fish. Moreover, previous studies demonstrated that exogenous protease could enhance the immunity of various aquatic species including Nile tilapia (Hassaan et al., 2020, Hassaan et al., 2021) and gibel carp (Xu et al., 2022).

In the present study, exogenous protease supplementation increased whole-body amino acid concentrations, possibly by improving the ADC of amino acids and enhancing their bioavailability. Amino acids serve as a key energy source and are essential for the growth and development of shrimp (Alam et al., 2004a). Arginine plays a crucial role in energy homeostasis in invertebrates. During periods of increased energy demand, arginine phosphate is converted to ATP and arginine through the activity of arginine kinase (Huang et al., 2020). In crustaceans, amino acids and their metabolites, particularly arginine, tryptophan and methionine, are crucial substrates for the immune system (Yang et al., 2019; Zhou et al., 2012). For instance, arginine serves as a precursor for immune system-related intermediates and metabolites such as nitric oxide (NO), creatine, polyamines and essential non-protein substances. NO plays a key role in immune cell proliferation, modulating antimicrobial effects and influencing various immune and antioxidant responses in host cells (Wu et al., 2009). Polyamines possess antitumor properties and promote T-cell biosynthesis (Hesterberg et al., 2018), whereas tryptophan is involved in diverse immune functions, including restoring oxidative damage, enhancing the phagocytic activity of hemocytes and reducing inflammatory cytokines (IL-1β, IL-6, IL-17 and TNF-α) (Kim et al., 2010; Yang et al., 2019). Numerous studies have demonstrated that amino acids and their metabolites play a pivotal role in immune-metabolic regulatory processes in various crustacean species (Alam et al., 2004b; Jin et al., 2015; Laranja et al., 2010). Therefore, in this study, enhanced amino acid bioavailability through increased proteolytic activity may have contributed to improved growth and cellular and humoral immune responses in shrimp.

Our findings demonstrated that exogenous enzyme supplementation in diets increased the intestinal villi length in shrimp. Furthermore, villi length exhibited a similar response to changes in nutrient availability, with higher nutrient ADCs showing higher villi length. The degradation of ANFs by exogenous proteases might have led to improved intestinal integrity and morphology in this study. Zuo et al. (2015) observed that the inclusion of exogenous protease in a soybean meal-based diet increased villi length and decreased serum diamine oxidase activity in weaned piglets. Increased diamine oxidase activity in the blood is a response to impaired intestinal integrity, mucosal maturation and allergic reactions caused by ANFs such as β-conglycinin (Luk et al., 1980). Similarly, several studies have explored the alteration of intestinal mucosal morphology upon the addition of proteases to animal diets, most of which have revealed the beneficial effects of exogenous protease supplementation (Adeoye et al., 2016; Cowieson and Roos, 2016; Ding et al., 2016; Xu et al., 2017).

In the present study, exogenous protease altered the gut microbiota of Pacific white shrimp. The colony counts of heterotrophic marine bacteria, Gram-positive bacteria and *Lactobacilli* spp. increased in response to dietary FM and exogenous protease, whereas the opposite result was observed for *Vibrio* spp. In aquatic species, the structure of the gut microbial community is mainly determined by dietary intake, nutrient absorption and digestive enzyme activity (Li et al., 2018; Tzuc et al., 2014). Exogenous enzymes can enhance probiotic function by providing appropriate substrates to promote the activity of the intestinal microbiota and the growth of beneficial microorganisms in the gastrointestinal tract (Bedford and Cowieson, 2012; Maas et al., 2021).

According to our findings, the dominant microbial species detected in the gut appeared to be related to protein and carbohydrate metabolism. For example, *Lactobacillus* spp., a widely known fermentation strain, possesses several unique metabolic characteristics, including the ability to produce acids and exopolysaccharides, hydrolyze protein and inhibit pathogenic bacteria (Dempsey and Corr, 2022). In particular, these bacteria produce antimicrobial compounds such as bacteriocin, lactic acid, organic acid and hydrogen peroxide, which can boost innate immunity (Pérez-Sánchez et al., 2014). In aquatic animals, the gut microbiota plays a vital role in nutrient absorption, improving immunity, enzyme production and amino acid and short-chain fatty acid generation, in addition to maintaining homeostasis (Egerton et al., 2018; Rajeev et al., 2021). In shrimp, the modulatory effect of dietary manipulation on gut microbiota has not been adequately demonstrated. Therefore, future studies should focus on a more detailed taxonomical evaluation of the effects of exogenous enzymes on the gut microbiota of *P. vannamei*.

## 5 Conclusion

The findings of this study demonstrated that protease supplementation in both HFM and LFM diets improved the growth, nutrient digestibility, feed utilization, immunity, immune-related gene expression, antioxidant capacity, digestive enzyme activity, gut microbiota and intestinal morphology of Pacific white shrimp. Notably, supplementation of the LFM diet with 800 mg/kg protease enhanced shrimp performance to levels comparable to those fed the HFM diet. Thus, 800 mg/kg protease effectively bridges the performance gap between LFM and HFM diets, demonstrating the ability of exogenous protease to enhance the nutritional quality and value of plant proteins in shrimp feed. These findings provide a cost-effective solution for the shrimp feed industry, enabling reduced reliance on FM while maintaining production efficiency and sustainability. Future studies are recommended to determine the effects of higher protease supplementation levels on the performance of shrimp.

## Credit Author Statement

**Mirasha Hasanthi:** Writing – original draft, Software, Investigation, Formal analysis, Data curation. **Rutchanee Chotikachinda:** Writing – review & editing, Resources, Conceptualization. **Nalin Medagoda:** Investigation, Formal analysis. **Kyeong-Jun Lee:** Writing – review & editing, Supervision, Methodology, Conceptualization.

## Data availability statement

All datasets analyzed in this study are available from the corresponding author upon reasonable request.

## Declaration of competing interest

We declare that we have no financial and personal relationships with other people or organizations that can inappropriately influence our work and there is no professional or other personal interest of any nature or kind in any product, service and/or company that could be construed as influencing the content of this paper.
